# Bibliometric and visualized analysis of myopic corneal refractive surgery research: from 1979 to 2022

**DOI:** 10.3389/fmed.2023.1141438

**Published:** 2023-07-28

**Authors:** Fang Yang, Yi Dong, Chen Bai, Mohammad Alzogool, Yan Wang

**Affiliations:** ^1^Clinical College of Ophthalmology, Tianjin Medical University, Tianjin, China; ^2^Department of Ophthalmology, Renmin Hospital, Hubei University of Medicine, Shiyan, China; ^3^Tianjin Eye Hospital, Tianjin Key Lab of Ophthalmology and Visual Science, Tianjin Eye Institute, Nankai University Affiliated Eye Hospital, Tianjin, China; ^4^Department of General Surgery, Taihe Hospital, Hubei University of Medicine, Shiyan, China; ^5^School of Medicine, Nankai University, Tianjin, China; ^6^Nankai Eye Institute, Nankai University, Tianjin, China

**Keywords:** bibliometrics analysis, corneal refractive surgery, global trends, VOSviewer, CiteSpace

## Abstract

**Background:**

Myopic corneal refractive surgery is one of the most prevalent ophthalmic procedures for correcting ametropia. This study aimed to perform a bibliometric analysis of research in the field of corneal refractive surgery over the past 40 years in order to describe the current international status and to identify most influential factors, while highlighting research hotspots.

**Methods:**

A bibliometric analysis based on the Web of Science Core Collection (WoSCC) was used to analyze the publication trends in research related to myopic corneal refractive surgery. VOSviewer v.1.6.10 was used to construct the knowledge map in order to visualize the publications, distribution of countries, international collaborations, author productivity, source journals, cited references, keywords, and research hotspots in this field.

**Results:**

A total of 4,680 publications on myopic corneal refractive surgery published between 1979 and 2022 were retrieved. The United States has published the most papers, with Emory University contributing to the most citations. The Journal of Cataract and Refractive Surgery published the greatest number of articles, and the top 10 cited references mainly focused on outcomes and wound healing in refractive surgery. Previous research emphasized “radial keratotomy (RK)” and excimer laser-associated operation methods. The keywords containing femtosecond (FS) laser associated with “small incision lenticule extraction (SMILE)” and its “safety” had higher burst strength, indicating a shift of operation methods and coinciding with the global trends in refractive surgery. The document citation network was clustered into five groups: (1) outcomes of refractive surgery: (2) preoperative examinations for refractive surgery were as follows: (3) complications of myopic corneal refractive surgery; (4) corneal wound healing and cytobiology research related to photorefractive laser keratotomy; and (5) biomechanics of myopic corneal refractive surgery.

**Conclusion:**

The bibliometric analysis in this study may provide scholars with valuable to information and help them better understand the global trends in myopic corneal refractive surgery research frontiers. Two stages of rapid development occurred around 1991 and 2013, shortly after the innovation of PRK and SMILE surgical techniques. The most cited articles mainly focused on corneal wound healing, clinical outcomes, ocular aberration, corneal ectasia, and corneal topography, representing the safety of the new techniques.

## 1. Introduction

Myopia is the leading cause of visual impairment worldwide (1). Thus, refractive surgery has become one of the most commonly performed ophthalmologic surgical procedures (2, 3). Over the last decades, the reliance on spectacles and contact lenses has decreased worldwide owing to the low risk and high success rates of refractive surgery (4). Since the cornea contributes to approximately two-thirds of the eye’s total optical power while being the most accessible part of the eye, myopic corneal refractive surgery is the mainstay of myopic correction. In 1896, Lendeer Jans Lans of Holland was the first to publish the idea of changing the cornea’s shape to correct refractive errors. In 1948, Jose Ignacio Barraquer Moner suggested that changing the corneal curvature could correct refractive error by sculpting corneal stromal tissue (5).

Radial keratotomy (RK) was first developed by the former Soviet scholar Svyatoslav N. Fyodrov in the 1970s (6) and was introduced in the United States in 1979. Since then, RK has been widely used as a treatment for myopia (7). Unfortunately, the initial surgical techniques were inherently imprecise, but the discovery of excimer laser photoablation in 1980 has brought precision to the process and has become the root of modern refractive surgery. The use of ocular excimer lasers was first reported in 1981 by Taboada, Mikesell, and Reed in United States (8). Stephen Trokel, a physical engineer and ophthalmologist, was the first to experiment with the use of an excimer laser on the cornea for refractive correction in 1983 (9). In 1985, Seiler performed the first procedure using an excimer laser on a sighted human eye (10). Subsequently, another technique known as *in situ* keratomileusis was developed in 1988 (11). In 1989, Ioannis Pallikaris from Greece was the first to perform laser *in situ* keratomileusis (LASIK) on a blind human eye at the University of Crete (12). Since then, there has been rapid progression in the use of excimer lasers for refractive and therapeutic purposes. Significant technological advances have led to refractive surgery becoming one of the fastest-evolving fields in healthcare. In recent years, clinical outcomes have improved remarkably due to advances in surgical methods and technology, such as laser epithelial keratomileusis (LASEK), femtosecond (FS) lasers, thin flap LASIK (sub-Bowman’s keratomileusis), enhanced laser delivery systems, improvement of eye trackers, and wavefront-guided and topography-guided lasers. Enhanced visual outcomes, refractive predictability, and low occurrence of complications, combined with these significant technical advances, have led to widespread acceptance of laser refractive surgery as a replacement for spectacles and contact lenses (10). A new technique called Small-incision lenticule extraction (SMILE) uses a FS laser to shape a refractive lenticule and then remove it through a minor wound. The potential advantages of SMILE over other techniques include lower laser energy requirements (13), less induction of higher-order aberrations (14), a significant decrease in corneal inflammation and keratocyte damage (15, 16), shorter duration and lower suction pressure during the procedure (17), greater tectonic strength, and a decreased occurrence of dry eye (18, 19).

Citation analysis is a widely used type of bibliometric method utilizing mathematical and statistical methods to explore and analyze the citation and reference patterns of both academic and scientific journals, papers, and authors. In this study, citation analysis was applied to study research impact, knowledge flows, and citation networks. Citation analysis has been used to visually highlight and provide a valuable overview of current academic literature, and to predict research trends (20). With the development of analysis software and the expansion of easily accessible online databases, citation analysis is gaining more and more attention worldwide. Although many studies on clinical outcome, efficacy, safety, stability, pathology, and biomechanical changes in surgery have been published, to our knowledge, the global research trend in myopic corneal refractive surgery has not yet been explored using bibliometric analysis. Therefore, this study aimed to investigate the prevailing status of myopic corneal refractive surgery research using Web of Science Core Collection (WoSCC) data. Bibliometric analysis was applied to analyze the existing international status of myopic corneal refractive surgery research, determine the highly influential factors in this field, and investigate hotspots in the research. Overall, this study provides not only a complete and extensive reference but also a promising reference for researchers.

## 2. Materials and methods

The publications were downloaded and extracted from the WoSCC provided by Thomson Reuters (Philadelphia, PA, United States). Data were acquired on August 21, 2022. The keywords used for the search were “myopic corneal refractive surgery” or some specific myopic corneal refractive surgery in the title or abstract because we were concerned with myopic corneal refractive surgery *per se* rather than related terminology. The search strategy was as follows: TI = “myopic corneal refractive surgery” or AB = “myopic corneal refractive surgery” or TI = “laser-assisted *in situ* keratomileusis” or AB = “laser-assisted *in situ* keratomileusis” or TI = “femtosecond laser-assisted LASIK” or AB = “femtosecond laser-assisted LASIK” or TI = “photorefractive keratectomy” or AB = “photorefractive keratectomy” or TI = “femtosecond laser-assisted LASIK” or AB = “femtosecond laser-assisted LASIK” or TI = “femotosecond lamellar extraction” or AB = “femotosecond lamellar extraction” OR TI = “epipolis laser *in situ* keratomileusis” OR AB = “epipolis laser *in situ* keratomileusis” or TI = “small incision lenticule extraction” or AB = “small incision lenticule extraction” or TI = “transepithelial photo-refractive keratectomy” or AB = “transepithelial photo-refractive keratectomy” or TI = “laser epithelial keratomileusis” or AB = “laser epithelial keratomileusis” or TI = “radial keratotomy” or AB = “radial keratotomy” or TI = “excimer laser refractive surgery” or AB = “excimer laser refractive surgery.” To increase accuracy, we restricted our search strategy to terms related to myopia in the title or abstract. The reason is that many of the reported publications were not associated with myopia when other search fields were used, such as keywords. As opposed to title, abstract, or keywords search queries, the title or abstract search is recommended in bibliometric studies because it significantly increases specificity with minimal loss of sensitivity.

Among the document types included were only articles and reviews (other types of documents, such as meeting abstracts, editorial materials, proceedings papers, and letters, were excluded). For the analysis, journal articles were used since they contain complete research ideas and results. Downloaded data from WoSCC are in “plain text” format, with “full records and cited references.”

The use of visualization software can create node-link maps that make it intuitive to observe publication outputs, hotspots, and other aspects of a research field. This study used VOSviewer v.1.6.10^1^ to analyze the data systematically. VOSviewer generated knowledge maps that represented items as nodes and links. There were nodes and labels corresponding to countries, organizations, authors, co-citation literature, and keywords based on their weights. Nodes are linked together based on their relationships. We used CiteSpace IV (Drexel University, Philadelphia, PA, United States) to identify keywords with strong citation bursts, which may indicate research frontiers. To evaluate the development of scientific research within a particular field, CitNetExplorer Software^2^ was used to visualize the citation networks and the connections between them. This software can perform cluster analysis and show the main research content of the research field.

## 3. Results

### 3.1. Yearly quantitative distribution of publications

**Figure 1:**
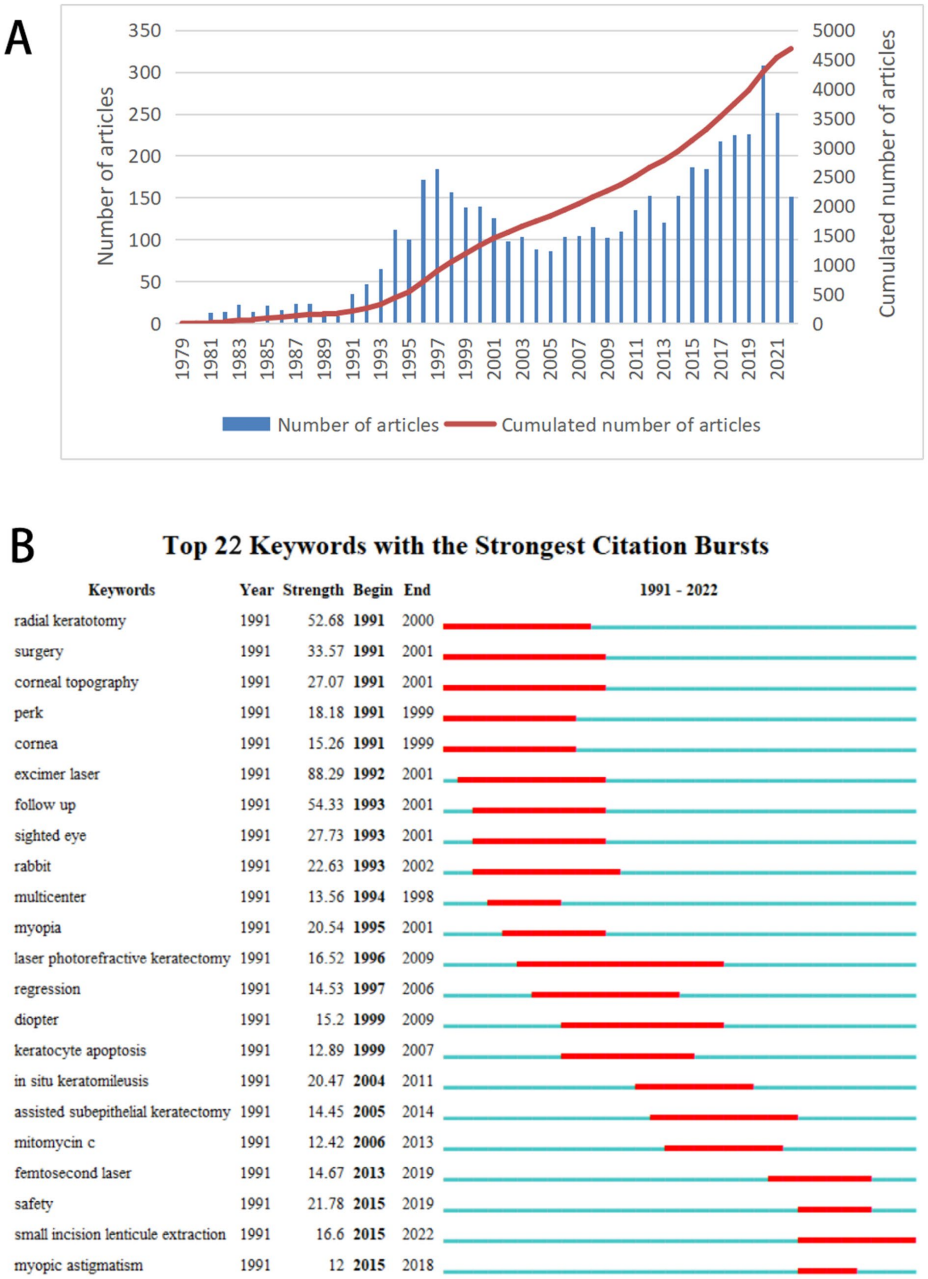
Annual publications and citation bursts analysis. **(A)** Number and Cumulated number of publications on myopic corneal refractive surgery per year (1979–2022). **(B)** Top 22 keywords with the strongest citation bursts on myopic corneal refractive surgery. The blue lines represent the base timeline, while the red segments represent the burst duration of the keywords.

The WoSCC database search resulted in a total of 4,680 articles. The first article on myopic corneal refractive surgery was published in 1979 (Figure 1A). In 2020, 308 articles were published, which was the highest yearly number (Figure 1A). In 2022, 152 articles had been published by August (Figure 1A), and the number is expected to rise significantly by the end of the year. We identified 22 keywords representing citation bursts by keyword burst detection analysis (Figure 1B). The first keyword was “radial keratotomy” detected in 1991 and lasted for 9 years. “Excimer laser” had the highest burst intensity (88.29) among the 22 keywords during the rapid development stage. “small incision lenticule extraction,” “femtosecond laser,” “safety,” and “myopic astigmatism” were the latest keywords in the rapid development stage.

### 3.2. Distribution of productive countries

The search results showed that 4,680 articles were found from 83 countries. As shown in Table 1, the top 10 cited countries involved in myopic corneal refractive surgery research published a total of 3,699 articles, accounting for 79.04% of the total number of publications. The United States had the largest number of publications (1,490 articles, 31.84%), followed by China (537 articles, 11.47%) and Germany (318 articles, 6.79%). The citation analysis showed that the United States had 41,020 citations, followed by Germany (7536) and the United Kingdom (6747).

**Table 1:** Top 10 cited countries in myopic corneal refractive surgery study.

Rank	Country	Number of citations (%)	Number of publications (%)
1	United States	41,020 (36.21%)	1,490 (31.84%)
2	Germany	7,536 (6.65%)	318 (6.79%)
3	England	6,747 (5.96%)	236 (5.04%)
4	China	5,506 (4.86%)	537 (11.47%)
5	Italy	5,455 (4.81%)	283 (6.05%)
6	Spain	4,769 (4.21%)	237 (5.06%)
7	France	3,989 (3.52%)	157 (3.35%)
8	South Korea	3,226 (2.85%)	184 (3.93%)
9	Brazil	2,986 (2.64%)	135 (2.88%)
10	Japan	2,969 (2.62%)	122 (2.61%)

**Figure 2:**
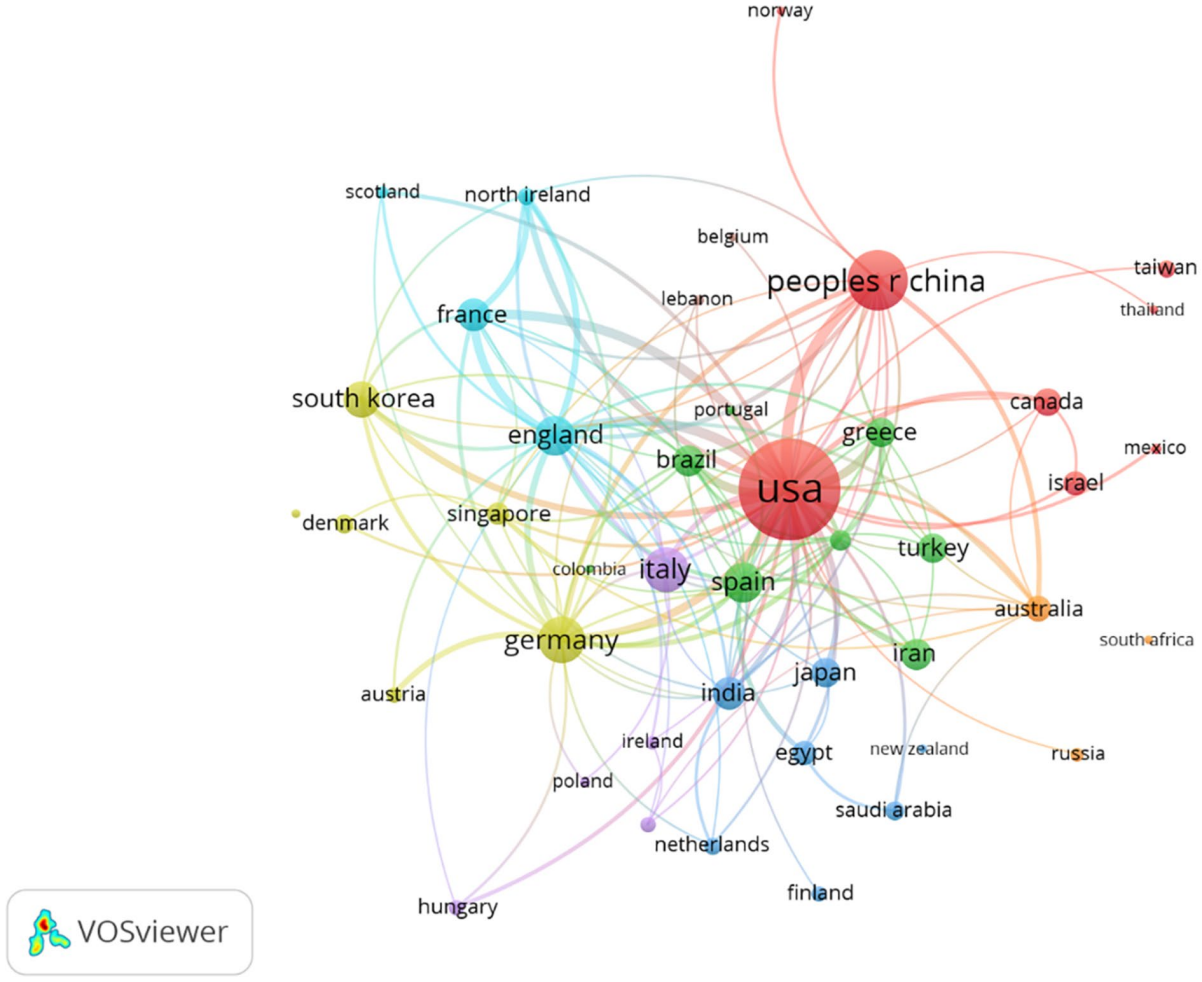
Network visualization map of countries’ collaboration in myopic corneal refractive surgery research. The size of the node represents the number of publications of the country and the thickness of the lines signifies the size of collaboration between the countries. The minimum number of documents of a country was set as 10. Of the 83 countries that were involved in myopic corneal refractive surgery research, 43 countries met the threshold.

The degree of communication between countries and the most influential countries in the field is reflected by country co-authorship analysis. The larger the node, the greater the country’s influence; the thickness and distance of the links between nodes define the strength of the cooperative relationships between countries. Figure 2 shows that the United States cooperated closely with numerous countries in the field of myopic corneal refractive surgery, including United Kingdom, China, France, Germany, and South Korea.

### 3.3. Distribution of prominent research organizations

**Figure 3:**
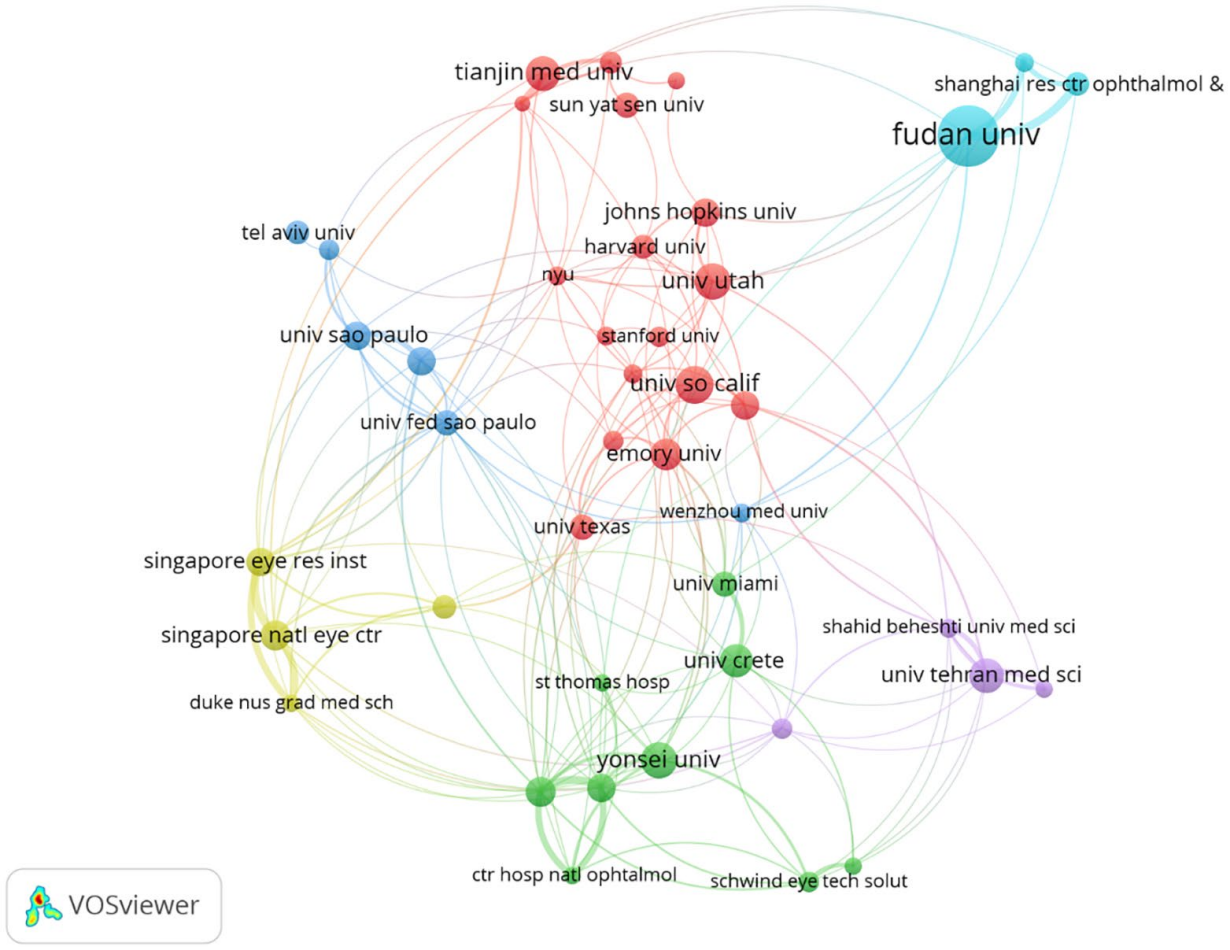
Network visualization map of main research organizations in myopic corneal refractive surgery study. The size of each node is determined by the number of publications from each institution. The width of each line represents the strength of links between institutions. The minimum number of documents of an organization was set as 30. Of the 3,084 organizations that were involved in myopic corneal refractive surgery research, 43 organizations met the threshold.

The search results showed that the top 10 cited institutions published 573 articles, accounting for 12.24% of the total number of publications (Table 2); seven of the 10 most cited institutions for myopic corneal refractive surgery were from the United States. Citation analysis showed that Emory University had 4,037 citations and was ranked first. The knowledge domain distribution map of myopic corneal refractive surgery research institutions is shown in Figure 3 and is based on co-authorship analysis. The size of each node corresponds to the number of published articles. The links between nodes represent collaborations. The stronger the node link, the closer the collaboration between the two organizations.

**Table 2:** Top 10 cited organizations in myopic corneal refractive surgery study.

Rank	Organizations	Country	Number of citations (%)	Number of publications (%)
1	Emory University	United States	4,073 (8.21%)	60 (2.93%)
2	University of Texas	United States	3,466 (6.99%)	46 (2.25%)
3	University of California	United States	2,713 (5.47%)	74 (3.62%)
4	Louisiana State University	United States	2,420 (4.88%)	38 (1.86%)
5	University of California Los Angeles	United States	2,199 (4.43%)	54 (2.64%)
6	Aarhus University	Denmark	2082 (4.20%)	43 (2.10%)
7	University of São Paulo	Brazil	1789 (3.61%)	54 (2.64%)
8	Harvard University	United States	1,694 (3.42%)	43 (2.10%)
9	Cleveland Clinic	United States	1,679 (3.39%)	37 (1.81%)
10	Fudan University	China	1,613 (3.25%)	124 (6.06%)

### 3.4. Distribution of authors and co-authorship of research groups

According to the search results, more than 20,728 authors have contributed to myopic corneal refractive surgery research. Table 3 lists the 10 most productive authors in myopic corneal refractive surgery. Among all authors, Zhou and Xingtao (104 publications) ranked first, followed by Wang and Yan (68 publications) and Reinstein and Dan Z (54 publications), reflecting their abundant contribution to the research of myopic corneal refractive surgery. The authors’ co-citation status was also analyzed. The results showed that Reinstein, DZ (1,430 co-citations), was the most commonly cited author, followed by Seiler, T (1,263 co-citations) and Wilson, SE (908 co-citations), indicating their relative influence on myopic corneal refractive surgery research.

**Table 3:** Top 10 productive authors and co-cited authors in myopic corneal refractive surgery study.

Rank	Author	Number of publications	Citation ratio	Co-cited author	Number of citations	Citation ratio
1	Zhou, Xingtao	104	13.73	Reinstein, DZ	1,430	26.48
2	Wang, Yan	68	12.81	Seiler, T	1,263	37.14
3	Reinstein, Dan Z	54	26.48	Wilson, SE	908	22.70
4	Moshirfar, Majid	52	10.46	Kanellopoulos, AJ	875	28.22
5	Archer, Timothy J	48	25.04	Alio, JL	803	25.90
6	Kymionis, George D	43	23.67	Sekundo, W	767	28.40
7	Jhanji, Vishal	42	7.86	Waring, GO	763	29.35
8	Mehta, Jodhbir S	42	24.71	Randleman, JB	586	22.63
9	Wilson, Steven E	40	22.70	Hersh, PS	547	26.05
10	Li, Meiyan	39	20.67	Gartry, DS	544	23.65

Citation ration = Number of citations/number of publications.

**Figure 4:**
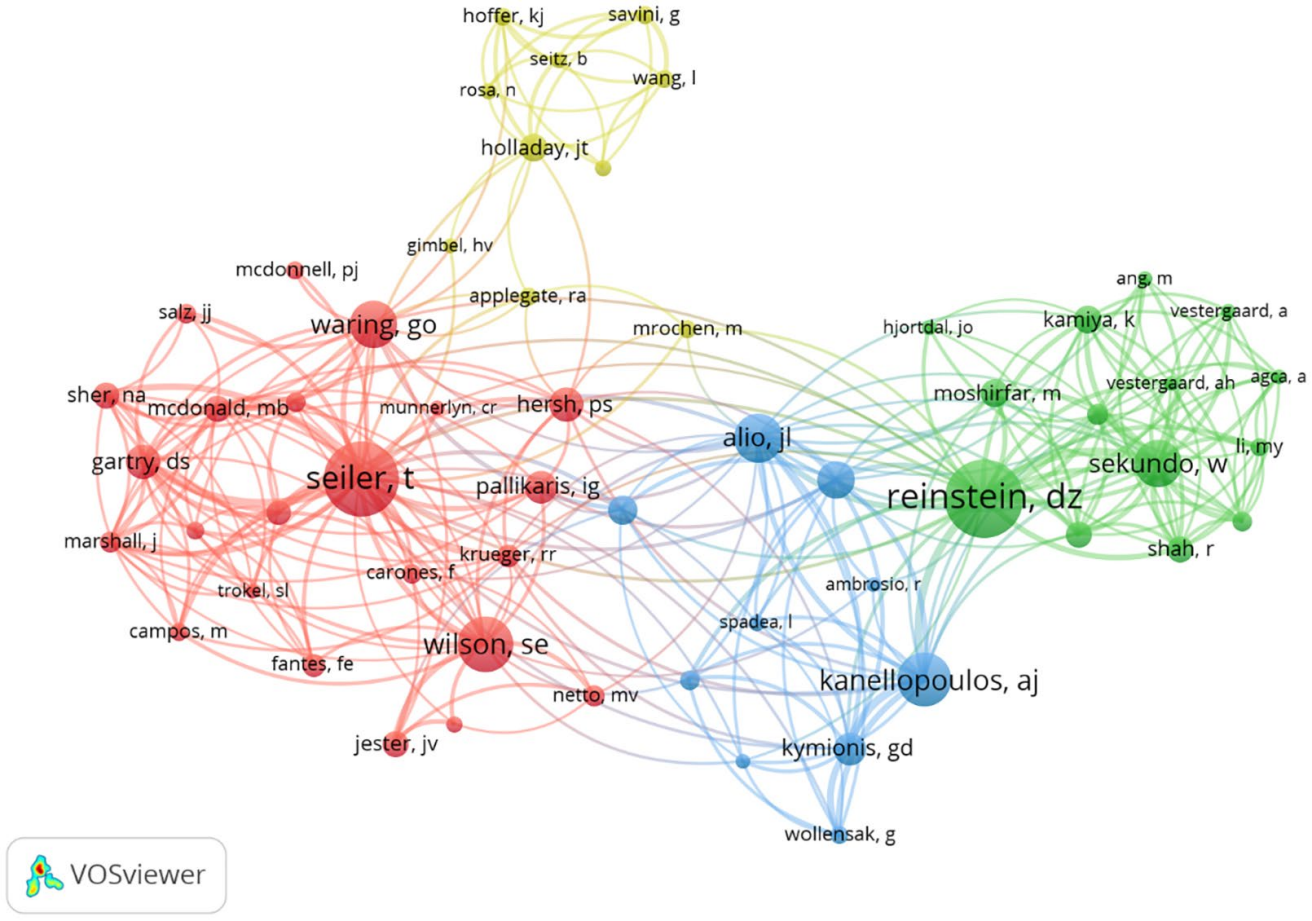
Co-cited authorship network in myopic corneal refractive surgery study. The size of the frame represents the number of publications of the author and the thickness of lines signifies the size of collaboration between the authors. The minimum number of documents of an author was set as 200. Of the 20,728 authors that were involved in myopic corneal refractive surgery research, 57 authors met the threshold.

Based on the co-authorship analysis, the knowledge domain map of authors of myopic corneal refractive surgery research is shown in Figure 4. There was a close collaboration between high-yielding authors, except for the group marked in yellow. Different co-authorship groups have different cores, such as the red group with Seiler T and the green group with Reinstein DZ as the core.

### 3.5. Top prolific source journals

Based on the retrieved results, articles on myopic corneal refractive surgery were published in 96 journals. Table 4 lists the top 10 journals with publications related to myopic corneal refractive surgery. According to the citations results, the citations of the *Journal of Cataract and Refractive Surgery* ranked first and the *Journal of Refractive Surgery* ranked second. The *Journal of Refractive Surgery* published the most significant number of articles (793, 16.94%), followed by the *Journal of Cataract and Refractive Surgery* (710, 15.17%) and *Ophthalmology* (237, 5.06%). These three journals accounted for 39.80% of the total number of articles published in this field. Considering the countries of publication, seven of the top 10 journals were from the United States, two were from the United Kingdom, and one was from the Netherlands.

**Table 4:** Top 10 cited source journals in myopic corneal refractive surgery study.

Rank	Journal	Publishing country	Citations (%)	Count (%)
1	Journal of Cataract and Refractive Surgery	United States	17,280 (18.87%)	710 (15.17%)
2	Journal of Refractive Surgery	United States	16,261 (17.76%)	793 (16.94%)
3	Ophthalmology	Netherlands	13,154 (14.37%)	237 (5.06%)
4	Archives of Ophthalmology	United States	6,144 (6.71%)	116 (2.48%)
5	American Journal of Ophthalmology	United States	5,718 (6.24%)	162 (3.46%)
6	Investigative Ophthalmology & Visual Science	United States	4,269 (4.66%)	80 (1.71%)
7	Cornea	United States	4,220 (4.61%)	263 (5.62%)
8	British Journal of Ophthalmology	United Kingdom	2,215 (2.42%)	70 (1.50%)
9	Experimental Eye Research	United States	1,632 (1.78%)	30 (0.64%)
10	Clinical Ophthalmology	United Kingdom	1,533 (1.67%)	134 (2.86%)

### 3.6. Top-cited publications

A myopic corneal refractive surgery research knowledge base can be efficiently constructed through the co-citation analysis of cited references. The minimum number of citations for a cited reference was set to 50 of the 34,267 cited references, and 136 met the threshold. Table 5 lists the top 10 co-cited references The top 10 papers were co-cited over 4,200 times, and the first was co-cited 570 times. The top 10 cited references primarily concentrated on outcomes and wound healing in refractive surgery.

**Table 5:** Top 10 co-cited references in myopic corneal refractive surgery research.

Rank	Title	Author	Year	Citations
1	Wound-healing after excimer laser keratomileusis (photorefractive keratectomy) in monkeys	Fantes, FE	1990	570
2	Photorefractive keratectomy-a technique for laser refractive surgery	Munnerlyn, CR	1988	529
3	Small incision corneal refractive surgery using the small incision lenticule extraction (SMILE) procedure for the correction of myopia and myopic astigmatism: results of a 6 month prospective study	Sekundo, Walter	2011	511
4	Risk assessment for ectasia after corneal refractive surgery	Randleman, J	2008	444
5	Results of small incision lenticule extraction: All-in-one femtosecond laser refractive surgery	Shah, Rupa	2011	397
6	Ocular aberrations before and after myopic corneal refractive surgery: LASIK-induced changes measured with laser ray tracing	Moreno-Barriuso, E	2001	365
7	Corneal stromal wound healing in refractive surgery: the role of myofibroblast	Jester, JV	1999	362
8	Excimer-laser *in-situ* keratomileusis and photorefractive keratectomy for correction of high myopia	Pallikaris, IG	1994	362
9	Myopic photorefractive keratectomy with the excimer laser - one-year follow-up	Seiler, T	1990	346
10	Comparison of corneal wavefront aberrations after photorefractive keratectomy and laser *in situ* keratomileusis	Oshika, T; Klyce, SD	1999	315

### 3.7. Myopic corneal refractive surgery research themes, frequent topics, and trends

**Figure 5:**
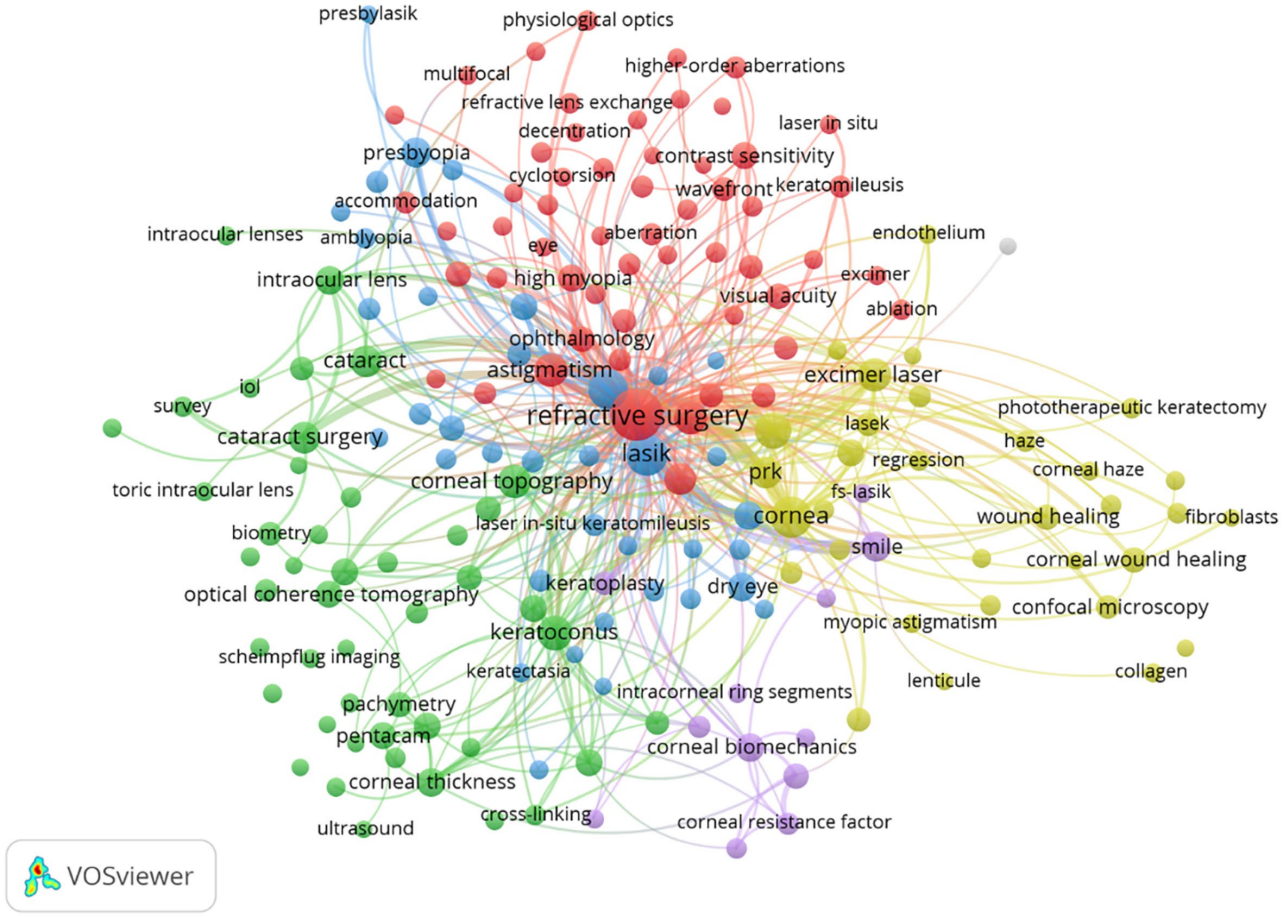
Co-occurrence network of keywords in myopic corneal refractive surgery study. The size of the points represents the frequency, and the keywords are grouped into five clusters: (Cluster 1 – Red) Outcome of refractive surgery; (Cluster 2 – Green) Preoperative examinations of refractive surgery; (Cluster 3 – Blue) Complications of myopic corneal refractive surgery; (Cluster 4 – Yellow) Corneal wound-healing and cytobiology research related to photorefractive laser keratotomy; and (Cluster 5 – Purple) biomechanics in myopic corneal refractive surgery. The minimum number of occurrences of a keyword was set as 8. Of the 4,133 keywords that were involved in myopic corneal refractive surgery research, 184 keywords met the threshold.

The research hotspots of myopic corneal refractive surgery have been identified by high-frequency keyword co-occurrence analysis the minimum number of occurrences of each keyword was set to eight. Of the 4,133 keywords related to myopic corneal refractive surgery research, 184 met the threshold. Keywords with similarity were clustered based on the network, and five major clusters were represented by red, green, yellow, purple, and blue (Figure 5), and Table 6 lists the top 10 keywords for each cluster.

**Table 6:** Co-occurence analysis of keywords.

Clustrer 1 (Red)	OF	Clustrer 2 (Green)	OF	Clustrer 3 (Blue)	OF	Clustrer 4 (Yellow)	OF	Clustrer 5 (Violet)	OF
Refractive surgery	731	Keratoconus	127	Lasik	278	Cornea	259	SMILE	65
Astigmatism	108	Corneal topography	94	Myopia	208	Photorefractive keratectomy	158	Corneal biomechanics	48
Femtosecond laser	103	Corneal thickness	88	Presbyopia	51	Excimer laser	95	Corneal hysteresis	30
Laser *in situ* keratomileusis	82	Glaucoma	78	Dry eye	41	PRK	89	Keratoplasty	26
High myopia	45	Intraocular pressure	55	Small incision lenticule extraction	39	Radial keratotomy	44	Corneal resistance factor	21
Contrast sensitivity	41	Optical coherence tomography	51	Hyperopia	39	Corneal wound healing	33	Corneal ectasia	19
Visual acuity	37	Pentacam	41	Refractive error	39	Wound healing	31	Ocular response analyzer	16
Ophthalmology	32	Central corneal thickness	39	Contact lenses	38	Confocal microscopy	27	Astigmatic keratotomy	10
Phakic intraocular lens	31	Keratometry	39	Contact lens	38	LASEK	25	Corvis St.	10
Laser	28	Pachymetry	39	Laser *in-situ* keratomileusis	32	Biomechanics	23	Intracorneal ring segments	10

Top 10 keywords in the 5 clusters. OF, occurrence frequency.

## 4. Discussion

The purpose of bibliometric analysis is to answer a specific research question based on a large number of publications (21) even without having access to special data collections, in contrast to systematic reviews, which attempt to address research questions based on a small number of publications (22, 23). Recently, bibliometric analysis has become widely recognized as an alternative tool for academically detailed information assessment in the information and library sciences. Although there have been many studies concerning myopic corneal refractive surgery, they were limited to a single aspect or had a short study period and did not include keyword bursts in their analyses (10, 18, 24, 25). In this study, we conducted a comprehensive bibliometric analysis of 4,680 available literatures concerning myopic corneal refractive surgery from 1979 to 2022; five groups were recognized in citation network, and keyword burst detection was carried out.

### 4.1. Global contribution to research on myopic corneal refractive surgery

A change in the number of academic papers is an important research index reflecting a growing trend in the relevant field. As shown in Figure 1A, 4,680 articles were retrieved for myopic corneal refractive surgery from 1979 to 2022, and the annual research output increased with time. The number of literatures on myopic corneal refractive surgery has been divided into four stages: the inception phase (from 1979 to 1991), the rapid development phase (from 1991 to 2001), the steady development phase (from 2001 to 2013), and another fast development phase (after 2013). At the start of the inception phase, RK was introduced in the United States in 1979 (7) and received widespread attention as a treatment option for myopia. The use of excimer lasers for refractive and therapeutic purposes has grown rapidly since the 1980s, with the advent of excimer lasers for photorefractive keratectomy (PRK) and LASIK. In 1990, Pallikaris and Buratto developed LASIK based on Barraquer’s lamellar incision practice 40 years earlier (26), which became a significant milestone and ushered in the first rapid development stage of myopic corneal refractive surgery. The first laser epithelial keratomileusis (LASEK) procedure was performed by Azar et al. in United States in 1996 (27). In 2000, the Food and Drug Administration (FDA) approved an FS laser for lamellar corneal surgery (28). Surgical techniques have evolved from FS lenticule extraction and pseudo SMILE to the most commonly used surgery, SMILE, which the FDA has approved for treating myopia and astigmatism (29). SMILE was first utilized by Sekundo and Blum in 2008 (30). The results of these inchoate studies were published in 2008 (30) and 2011 (31). Subsequently, the number of articles published annually increased. As of 2017, more than a million SMILE procedures had been performed worldwide (32).

International cooperation among countries in scientific research is becoming increasingly important. As shown in Table 1, the United States ranked first in the number of publications and citations, at 31.84 and 36.21%, respectively. Meanwhile, China contributed 537 articles, accounting for 11.47% of publications. At the same time, the United States and China teamed up intensely with many other countries in the myopic corneal refractive surgery field, as shown in Figure 2; the United States and China are international scientific centers of myopic corneal refractive surgery research and play an important role in academic exchanges and cooperation. Adequate funding, the sharing of advanced techniques, and equipment settings are also essential. The most productive organizations and collaboration within the groups can be recognized and are shown in Table 2; the most influential research institute was Fudan University (124 documents), followed by the University of California (74 documents), and Emory University (60 documents). According to the citation analysis, Emory University had 4,037 citations and was ranked first. These organizations are at the heart of the research network. Among all authors, Zhou Xingtao contributed 104 publications and was ranked first, followed by Wang Yan, the first surgeon to report the clinical results of SMILE surgery in China (33). Information on author co-citations was also analyzed. Regarding authors’ analysis, Reinstein DZ was the most cited author, and Seiler T had the highest citation ratio.

Valuable information for researchers seeking collaboration opportunities can be provided by establishing a co-authorship network knowledge map. Figure 4 shows the co-authorship groups. The red-colored team includes Professor Seiler who first applied excimer lasers to the human eye in 1985 (10). Seiler performed the first wavefront-guided myopic corneal refractive surgery putting forward the concept of personalized cutting as the focus. The green-colored team centers on Professor Reinstein, a pioneer in corneal measurement techniques and instruments, who invented laser-blended vision therapy to treat the reading vision of patients with presbyopia. The yellow-colored team centers on Professor Holladay, the inventor of Holladay’s formula. The blue-colored team has Professor Alio as the focus.

A distribution analysis of academic journals helps to identify the core journals in particular field of research. We found that the *Journal of Refractive Surgery* has published the maximum number of articles, whereas, the *Journal of Cataract and Refractive Surgery* has the highest number of citations and is most influential.

### 4.2. Intellectual base

We used citation parameters to describe relevant topics in the selected articles on the premise that high-quality research will be widely cited. Through co-citation analysis, a large number of cited references can effectively display the research background. As shown in Table 5, the top-cited article is “Wound-healing after excimer laser keratomileusis (PRK) in monkeys,” published in 1990 in the *Archives of Ophthalmology* with 544 citations. This study deduced that the monkey anterior cornea showed a mild, typical wound-healing response after an excimer laser keratomileusis procedure (34). The second top-cited article is “PRK-a technique for laser refractive surgery,” published in 1988 in the *Journal of Cataract and Refractive Surgery* with 512 citations. In this paper (35), the conditions for PRK, which utilizes tissue ablation with far-ultra-violet radiation to directly reshape the central optical zone of the cornea, were described, and the equations for the tissue ablation to accomplish the required refractive corrections were presented. The third top-cited article is “Small incision myopic corneal refractive surgery using the SMILE procedure for the correction of myopia and myopic astigmatism: results of a six-month prospective study,” published in 2011 in the *British Journal of Ophthalmology* with 456 citations. This study suggests that SMILE is a hopeful new flapless and minimally invasive technique for correcting myopia and myopic astigmatism (31).

### 4.3. Focus on myopic corneal refractive surgery

Keyword co-occurrence analysis can be used to represent the search topic and reveal the internal structure of the involved literatures and frontal subjects. As given in Table 6, the themes of corneal refractive surgery mainly formed five clusters. Combined with the characteristics and present status of corneal refractive surgery research, the following five groups are discussed:

#### 4.3.1. Outcome of myopic corneal refractive surgery

In recent years, with the accumulation of experience and continuous improvements in technology, the safety and efficacy of refractive surgery have significantly improved. RK can be used to treat myopia, but it has been replaced by corneal laser surgery due to its lower safety and fecklessness (10). With the application of excimer lasers for myopic corneal refractive surgery, the effectiveness, accuracy, security, predictability, and stability of refractive surgery have been greatly improved. A review (36) of nearly 100 studies published since 2008 revealed that up to 99.5% of patients who underwent laser refractive surgery had uncorrected distance vision better than 20/40. 98.6% of these patients had a refractive target within ±1.0 diopter, and almost 98.8% of patients were satisfied with their results. Seiler and Wollensak (37) reported that PRK is an effective technique to rectify myopia by up to −7.0 D. Nevertheless, corneal haze and myopic regression may usually appear after PRK procedure (38). Although improvement in uncorrected visual acuity is faster with LASIK than with PRK, the long-term effectiveness outcomes are generally similar between the two procedures (39). LASIK is more efficient, safe, and stable than PRK in the correction of higher myopia and creates less postoperative corneal haze (40, 41). Furthermore, the results of LASIK surgery are three times more predictable than PRK (40). In a meta-analysis, LASEK-treated eyes showed no significant benefit over PRK-treated eyes in terms of clinical outcome (42). All-in-one FS refractive correction is safer, predictable, and is more efficient in treating myopia and myopic astigmatism due to its small incision technique (43). SMILE also achieved similar effectiveness, predictability, and safety in comparison to FS-LASIK (44). Corneal biomechanical strength and corneal nerve protection were significantly improved with SMILE compared to LASIK (45) or PRK (44). However, this technique lacks automated centration and cyclotorsion control, so several concerns have been raised regarding its capability to correct moderate or high levels of astigmatism (46).

Additionally, a short-tear breakup time-type dry eye may appear in patients treated with SMILE (47), while the postoperative dry eye syndrome incidence was lower in SMILE than in FS-LASIK (44). A network meta-analysis compared the postoperative efficiency, predictability, safety, and visual quality of all dominating modes of laser corneal refractive surgeries for correcting myopia in 2017. These procedures can be broadly divided into 3 categories: corneal surface ablation surgery, corneal stromal ablation surgery, and refractive corneal lenticule extraction. Surface ablation procedures include photorefractive keratectomy (PRK), transepithelial photorefractive keratectomy (T-PRK), laser epithelial keratomileusis (LASEK), and epipolis laser *in situ* keratomileusis (Epi-LASIK) (48). Corneal stromal ablation procedures include laser *in situ* keratomileusis (LASIK) with the flap created with either a mechanical microkeratome or femtosecond-based microkeratome (FS-LASIK). Refractive corneal lenticule extraction procedures include femtosecond lenticule extraction (FLEx) and small incision lenticule extraction (SMILE) (49). No statistically significant differences in either visual outcomes or visual quality between FS-LASIK, LASIK, Epi-LASIK, PRK, T-PRK, LASEK, FLEx and SMILE were found (50). However, in predictability of outcome, FS-LASIK performed better than other surgeries (50).

Patients who have undergone refractive surgery usually have some visual complaints even if their visual acuity was 20/20. The human eye normally has a substantial number of aberrations (51). It was shown that high-order aberrations (HOAs) increased after surgery, and a high correlation was revealed between HOAs, corneal spherical aberration, and visual performance (52, 53). The total wavefront error increased by a factor of 17.65 on average in any treated individual (54). Correspondingly, refractive surgery can induce a mass of third- and higher-order aberrations, with spherical aberrations increasing the most (52). Wavefront aberrations of the cornea were increased and the relative contribution of coma-and spherical-like aberrations was changed in PRK and LASIK procedures (55). In Wen’s study (50), LASIK appeared to induce a higher degree of HOAs than other surgical methods, regardless of pupil size, and the surface group (PRK, T-PRK and LASEK) was better than the stromal group (FS-LASIK, LASIK and Epi-LASIK), especially for 6-mm pupils. Previous research showed that optimal ablation algorithms and procedures are essential to avoiding new aberrations and eliminating existing high-order aberrations while obtaining the desired correction of refractive error.

#### 4.3.2. Preoperative and postoperative examinations of myopic corneal refractive surgery

Refractive surgery aims to obtain good postoperative visual acuity and patient satisfaction. Therefore, effective patient selection is essential. A thorough preoperative examination and evaluation must be performed with full consideration of the motivation and expectations of the patient as well as the possibilities, complications, and contraindications of each operative technique. Routine procedures for myopic corneal refractive surgery include measurement of intraocular pressure (IOP), vision acuity, slit-lamp examination, fundus examination, dry eye screening, refractive status examination, corneal curvature and corneal topography, wavefront aberration, and ocular ultrasound. Randleman et al., using retrospective comparative and case–control studies, reported that the risk factors for corneal ectasia after myopic corneal refractive surgery included topographic abnormalities, low preoperative corneal thickness, inadequate residual stromal bed thickness, high myopia, and young in age (56). Other factors may also predict postoperative corneal ectasia, such as erratic refractions, eye rubbing history, a family history of ectatic corneal disease, and an underlying increase in corneal elasticity (56). Nevertheless, unacknowledged preoperative corneal ectasia is another major risk factor of postoperative corneal ectasia (57). Ectasia can occur after an otherwise uncomplicated laser *in situ* keratomileusis procedure, even in the absence of apparent preoperative risk factors (58).

Additionally, it is necessary to eliminate early keratoconus or forme fruste keratoconus before surgery, as rapidly progressive corneal ectasia can occur in such cases (59). Keratoconus is a non-inflammatory disease of corneal thinning characterized by anterior protrusion of the cornea and stromal attenuation (60). Since the first report of keratectasia in 1998 (61), many cases have been reported. In some situations, ignoring topographic abnormalities will result in serious consequences. For example, LASIK or PRK performed in the eye when true keratoconus or early keratoconus is present but not detected can lead to severe corneal damage and result in progressive corneal thinning and eventual need for corneal transplantation (62). Thus, detecting keratoconus is essential to avoid unpredictable results in refractive surgery.

The most challenging determinations clinicians must make involve distinguishing true early keratoconus from similar patterns, such as contact lens-induced warpage or normal corneas with asymmetric bowtie. Corneal topography is one of the most sensitive methods for detecting early keratoconus (63, 64). Topography can often provide characteristic clues to the presence of this disease before the cornea becomes significant thinner or other signs appear, such as abnormalities detectable by slit-lamp biometrics or light reflection under funduscopy. Unlike corneal topographers, tomographers generate a three-dimensional recreation of the anterior segment and provide information about the corneal thickness, which can evaluate the whole cornea by obtaining information from both anterior and posterior corneal surfaces (65). Progression is one of the most important markers of true keratoconus (62). In case of uncertainty (e.g., when considering whether to proceed with myopic corneal refractive surgery), the best course of action is to follow the topography and other indicators, such as central corneal thickness, over at least a year. Increased steepening over time is highly suggestive of true keratoconus (62). In recent years, machine learning has been applied to the detection of keratoconus (66) and in the analysis of corneal images (67, 68). Furthermore, corneal biomechanics have been employed for keratoconus detection. Wang et al. provided a new potential approach for diagnosing keratoconus solely from the perspective of corneal biomechanics (69). Without corneal topographic examination, machine learning makes rapid and accurate keratoconus diagnosis possible (69).

Determining the corneal thickness is a prerequisite for avoiding complications from refractive surgical procedures. Accurate measurement of corneal thickness helps to detect and manage corneal pathology associated with corneal thinning and to distinguish keratoconus from contact lens-induced corneal thinning (70); this allows the surgeon to calculate the depth of the residual corneal tissue and determine the safe limits for a given procedure (71). Preoperatively, the corneal thickness measurement was equivalent using Orbscan II, conventional ultrasound, or confocal techniques, but ultrasound biomicroscopy readings thicker (72). Pentacam was more accurate than Orbscan II, especially after refractive surgery (73).

In refractive surgery, pupil size is considered to be an essential factor of optical quality. Numerous of studies have analyzed the connection between the ablation zone and mesovisual pupil size in night vision problems after laser correction (74–77). Therefore, it is advisable to accurately measure preoperative pupil diameter under low-light illumination conditions. The most commonly used devices for scotopic and low-mesopic pupil size measurements are handheld infrared pupillometer (Colvard, Oasis, CA) and digital pupillometer(Procyon, United Kingdom), which allow coinstantaneous or close measurements (78).

It is common for patients to have lower IOP after corneal refractive surgery than before. The IOP measured after PRK and LASIK for myopia may be reduced because of reduced corneal thickness and curvature and, possibly, tissue softening after natural healing (79). The IOP in the central part of the cornea after PRK is lower than that in the corneal periphery (80). The reduction in measured IOP following refractive surgery, by about 0.5 mmHg/D of myopic correction, needs to be remembered when possible abnormality of IOP in such patients is being considered (79). Meanwhile, IOP is influenced by body position and different devices (81). It was shown that IOP increase in the supine and standing positions (81).

IOL power calculation after corneal refractive surgery is important and challenging due to inaccurate measurement of anterior keratometry, keratometric index variation, and wrong effective lens position (ELP) estimation (82, 83). An Advanced Lens Measurement Approach (ALMA) method (84) which was published by Rosa et al. can improve R Factor (85) and ALxK (86).

#### 4.3.3. Complications of myopic corneal refractive surgery

Laser refractive corneal surgery is a common operation with a low complication rate (38). However, because this is an elective procedure that improves the quality of life by restoring uncorrected visual acuity, any adverse events could seriously affect patient satisfaction. Patients who experience halos, increased glare, irregular astigmatism, corneal scarring or residual ametropia, are often unsatisfied (42). Dry eye is another common side effect due to reduced tear secretion from nerve damage and inflammation of the cornea. Fortunately, dry eye is generally an interim problem and can be treated effectively with lubricating eye drops or other methods. However, without proper treatment, pre-existing dry eye can get worsen (87). Iatrogenic keratectasia is one of the most severe complications. Rarely, this procedure weakens the corneal biomechanical strength, and results in corneal ectasia (88). Since the first report in 1998 (89), the characteristics of patients who developed corneal ectasia after surgery in a small case series were reported (58, 90–106).

There are few postoperative complications with PRK except for corrections greater than six diopters (107). Typically, a corneal haze appears in the first month after surgery and is the most severe at three to 6 months, after which it gradually decreases (108). Corneal haze continus to improve during long-term follow-up (108). At 12 months postoperatively, 3% of patients had haze, and 3.6% reported glare or halos (109). Ptosis occurred in 0.4% of eyes, and intraocular pressure increased significantly in 3.5% of eyes due to corticosteroid treatment (109). During the 18-year follow-up period, there was no evidence of a progressive hyperopic metastasis, corneal ectasia, or late-onset corneal haze (108).

Complications of LASIK in the papers published in peer-reviewed journals included night vision problem (14.0%), haze (8.7%), reoperation (8.2%), interface debris (6.8%), wrinkle (5.9%), central island (5.3%), induced astigmatism (5.1%), free cap (4.9%), decentration (4.7%), epithelial ingrowth (4.3%), irregular flap (4.0%), short flap (3.0%), perforated lenticule (2.6%), incomplete cut (2.5%) and sliding flap (1.4%) (110). Flap displacement (111), diffuse lamellar keratitis (112), and epithelial ingrowth (113) are flap-related complications, all of which can be treated with topical eye drops or, in rare cases, by relifting the flap.

SMILE has some advantages, including the absence of a corneal flap and better corneal biomechanical stability (114). However, some intraoperative complications, such as incisional bleeding, suction loss, subconjunctival hemorrhage, opaque bubble layer, black areas, lenticule tearing, unintended posterior plane dissection and incision abrasion (115), are observed in some cases. Although intraoperative complications inevitably occur, patients may achieve satisfactory visual outcomes although intraoperative complications inevitably occur, with appropriate management techniques (115). Some of the postoperative complications, including diffuse lamellar keratitis (DLK), punctate epithelial erosions, corneal infiltrates, foreign interface body, interface debris/secretion, interface haze, corneal edema, corneal striae and epithelial ingrowth, may temporarily affect visual recovery; however, most are resolved with appropriate management (116). Irregular corneal topography occurred in 1.0% of eyes, leading to ghost images or reduced corrected distance visual acuity at 3 months or ghost images (117).

#### 4.3.4. Corneal wound healing and cytobiology research related to myopic corneal refractive surgery

As shown in Table 5, the top-cited articles concerning wound healing after excimer laser keratomileusis. The predictability and stability of refractive surgery were undermined by wound-healing properties of the cornea, which lead to discrepancies between attempted and achieved visual outcomes after PRK, LASIK, and other keratorefractive procedures (118). With extensive research on wound healing after myopic corneal refractive surgery, achieved by the mechanical responses of the cornea to injury and dominated by the stroma, awareness of its role in refractive surgery has developed. Any mechanical or biological response to injury influences optical properties; in some cases, a tendency to mechanical instability or abnormal healing regulation can result in severe complications such as corneal ectasia or loss of corneal clarity. Variations in wound healing are the underlying cause of the varied refractive outcomes and progressive effect of incisional keratotomy (119–121), and these variations determine regression and complications following PRK (122, 123).

The wound healing process begins with epithelial injury, which may take the form of microknife, femtosecond laser disruption, mechanical scratching, or alcohol exposure. Subsequently, damaged epithelium cells and epithelial basement membrane release cytokines, including tumor necrosis factor-alpha (TNF-α), interleukin (IL)-1 (124), bone morphogenic proteins two and four, platelet-derived growth factor (PDGF), and epidermal growth factor (EGF) (125). These factors, along with others derived from the tears, trigger various responses in underlying stromal keratocytes. The keratin Fas ligand binds to the Fas receptor on nearby keratocytes and leads to apoptosis (124). After this, additional cells experience the pro-inflammatory process of necrosis (126). The remaining keratocytes begin to proliferate and migrate within 12–24 h, producing activated keratocytes, fibroblasts, and possibly myofibroblasts responsible for repopulating the depleted stroma (127). In addition, pro-inflammatory chemokines of epithelial cells or keratocytes respond to IL-1 and TNF-α within 24 h of injury, triggering stromal infiltration of T cells, macrophages/monocytes, and polymorphonuclear cells. These cells arrive via the limbal blood supply and tear film, and then, play a part in the phagocytosis of apoptotic and necrotic debris and possibly serve other functions in the stroma (128). Myofibroblasts stained with antibodies against alpha-smooth muscle actin (α-SMA) can be observed in the anterior stroma directly below the damaged areas of epithelial basement membrane one to 2 weeks after injury, depending on the surface irregularity, correction level, and other factors (129). Owing to altered corneal crystalline production, these cells revealed reduced transparency and play a comprehensive role in collagen and extracellular matrix remodeling through the production of collagen, collagenases, matrix metalloproteinases, gelatinases and glycosaminoglycans (130). The appearance of myofibroblasts is associated with a significant increase in a stromal haze (122, 123). The anterior cornea of monkey eyes after excimer laser keratomileusis showed a mild, typical wound healing response (35). Marshall et al. discovered that all corneas were clear immediately after 3 mm diameter discs were excided from the optical zone of the monkey corneas using an excimer laser at 193 nm at various depths up to 130 μm, except for the deepest ablation (131). The haze gradually resolved over 6 months, but the deepest discs were still identifiable on slit-lamp examination (131). The morphology was nearly normal at 8 months, except for the absence of Bowman’s membrane and the immediate subepithelial stromal fibers that still had some degree of disorder (131).

Nevertheless, there are important differences in the speed, intensity, and spatial distribution of the wound-healing activity among different surgical approaches of laser vision correction. Extensive injury and removal of the epithelial cells, epithelial basement membrane, Bowman’s layer, and part of the anterior stroma were involved in PRK; these structures were left relatively undisturbed in LASIK, except at the edge of the flap under the stromal-epithelial flap (118). The essential determinants of corneal wound healing may be the level and distribution of keratocyte apoptosis and subsequent activation of stromal keratocytes regeneration, which were associated with variability and regression after PRK and LASIK (128). This difference in the degree of central epithelial injury is a major factor in the clinical and histological differences observed after PRK and LASIK. The wound healing response in PRK was amplified and higher rates of regression and haze were generated owing to the destruction of the central corneal epithelial basement membrane. The haze development after PRK is directly related to increased cell reflectance of many wound-healing keratocytes (132). Apoptosis and proliferation of keratocyte and myofibroblast generation were observed significantly greater after PRK for high myopia (-9D) than after LASIK for equivalent myopia in rabbit corneas (133). Wound-healing responses may be more obvious after FS flap creation than after mechanical microknife surgery. Due to the fulminic cavitation procedure related with plasma formation, the interface irregularity may be higher after FS flap formation (134, 135), while, DLK and flap-edge DLK increased (136). Fewer cellular ultrastructural changes have been observed after SMILE (137). Except for the side-cut incision, the corneas did not display any opacity at any time (138). Unlike the PRK surgery, no epithelial and endothelial cell damage was observed after SMILE (137). Corneal stromal wound healing after SMILE and FS-LASIK was almost identical to keratocyte proliferation and apoptosis in a human donor eye model (139). Nonetheless, Liu et al. found that SMILE induced significantly less acute inflammation in the cornea and aqueous humor in rabbit models than FS-LASIK (140). However, reactive fibrosis near the laser application site was less pronounced after SMILE, and the surface texture of stromal bed was smoother after LASIK (139).

#### 4.3.5. Biomechanics in myopic corneal refractive surgery

Biomechanics has also been a concern in recent years. Morphological changes and thinning of the cornea after refractive surgery may affect corneal biomechanics. Accordingly, linking the morphology of the cornea to its mechanical behavior is crucial to understanding its mechanical properties. The cornea is defined as a complex anisotropic composite with biomechanical properties characterized by highly nonlinear elasticity and viscoelasticity. In terms of corneal biomechanical behavior, several structural features of the cornea have been speculated or proven to play an essential role in corneal biomechanical behavior. The stroma accounts for more than 90% of corneal thickness and controls corneal biomechanics (141). In particular, the anterior stroma consists of dense, regularly packed interwoven collagen lamellae known to be inserted vertically into Bowman’s layer. The anterior stroma is the strongest part of the cornea due to the high tensile strength provided by this collagen network structure, making it 50% stiffer compared to the mid or posterior stroma (135). The anterior stroma is also resistant to swelling owing to its structure, enabling the preservation of corneal curvature (142). However, corneal refractive surgery not only gives a corneal flattening, thinning and biomechanical changes, but also induces anterior chamber depth (ACD) and axial length (AL) decrease (143, 144).

Worldwide, more than 4 million people undergo elective refractive surgery each year to correct their vision; Studies have shown that postoperative corneal ectasia occurs in 0.04–0.6% of patients undergoing refractive surgery, and that, LASIK accounts for 96% of all cases (130). However, with this low incidence rate, the safety of refractive surgeries is relatively high. Therefore, understanding and utilizing corneal biomechanical properties to evaluate the suitability of patients for myopic corneal refractive surgery can help to predict its outcome and avoid complications, as well as help to improve the efficacy and safety of the surgery. Keratoconus is associated with corneal biomechanical abnormalities. These abnormalities lead to progressive thinning of the cornea and focal curvature changes in the cornea, due to loss of the collagen matrix and surrounding components, resulting in reduced biomechanical integrity (145). In most cases, the disease must progress into advanced stages that affect vision before it can be diagnosed by relying on changes in thickness, topography, and other morphological features. Therefore, it is desirable to look for other methods to aid in the diagnosis before these changes occur, and one of the most effective methods is to identify biomechanical abnormalities.

Corneal biomechanics can be affected by external and environmental stimuli such as variety of injuries and surgeries, different hydration statuses, and hypoxia. Studies have shown that the clinical manifestations of these biomechanical changes are immediate corneal shape changes, shape instability over time and increased sensitivity to shape changes (118). Notably, the same study indicated that the discrepancy between the expected outcomes after corneal refractive surgeries and the actual outcomes achieved after these surgeries is due to the biomechanical properties and wound healing properties of the cornea, which clearly impairs the predictability and stability of surgery (118). These patients had corneal biomechanical abnormalities before surgery, which manifested as subclinical keratoconus (145). In any refractive surgical procedure involving corneal surface ablation such as LASIK and PRK, the corneal lamellae are immediately transected in a circumferential direction. It is important to determine that the lamellar tension in the remaining peripheral lamellar segments is reduced due to central ablation, thereby reducing the local resistance to swelling, and resulting in peripheral stromal thickening (146). At the margin of the ablation zone, centripetal stress may be generated in the underlying lamellae through the dense interlaminar connections caused by peripheral stroma expansion (146). Empirically, hyperopic responses predominate when ablation is limited to the anterior stroma, whereas gradually deepening circumferential damage leads to corneal steepening (147, 148).

Regarding changes in the biomechanical strength of the cornea, a number of studies have been conducted to compare these changes after flap lifting procedures and intrastromal flapless procedures (149, 150). It is noteworthy that different studies have found a wide difference in biomechanical effects between individuals (151). Compared to other procedures, the anterior stroma contains much more collagen fibers during LASIK. Models predict that LASIK results in a 55–65% reduction in corneal elasticity (152), whereas PRK results in about a 20% reduction (153). A study by Hassan et al. comparing some biomechanical parameters measured with corneal visualization Scheimpflug technology (CorVis ST) before and after LASIK and PRK procedure has reported significant changes in the early postoperative period. However, there were no significant differences between most of the biomechanical parameters preoperatively and 1 month after the surgeries (154). SMILE may lower the risk of complications compared to LASIK, and has been shown to have similar outcomes to LASIK while tending to provide advantages regarding epithelium maintenance. Furthermore, SMILE has biomechanical advantages over LASIK due to its smaller incision size and reduced number of collagen fibers involved in the cutting process (145). In addition, further benefits derive from the removal of tissue deep within the stroma, which helps to maintain a strong anterior corneal stroma and Bowman’s layer (145). A previous study reported a 49% mean reduction in the stiffness of stromal collagen fibers in the flap area after flap-based procedures. Loading increase effects were observed in flap-based cases and SMILE cases, and results showed lower stromal bed displacements and stresses in SMILE cases.

## 5. Strengths and limitations

The present study is the first bibliometric analysis of myopic corneal refractive surgery performed using the literature from the 1970s. To acquire deep insight into myopic corneal refractive surgery research, VOSviewer was used to identify the hotspots and major clusters in this field. But this study has some limitations. First, the publications were extracted from the WoSCC from 1979 to 2022, which may not sufficiently represent all myopic corneal refractive surgery research topics. Second, because most publications in WoSCC were in English, a linguistic bias may exist. Third, the collaboration network analysis successfully displayed the co-occurrence (distance between the two nodes/items) and the institutions’ co-authorship (the links’ strength). But bibliometrics software could not distinguish the real author contribution among complicated partnership, which requires researchers to read the original literature themselves. Finally, though analysis was done by software objectively, the way to interpret these results will have inherent subjective bias by individuals.

## 6. Conclusion

A scientific map of myopic corneal refractive surgery research was constructed, including annual publications, national distribution, international cooperation, author publications, source journals, cited articles, and keywords. The results of this study can provide a reference for ophthalmologists to choose appropriate journals for publication, institutions, or authors for collaboration. The extracted keywords allow researchers to identify new topics and help predict research directions.

Based on the bibliometric analysis, myopic corneal refractive surgery had two rapid development stages, from 1991 to 2001 and after 2013. United States and China are the international scientific centers of myopic corneal refractive surgery research. The most productive institution was Fudan University. The most cited institution was Emory University. Reinstein DZ, Seiler T, Zhou XT, and Wang Y are the key researchers in this field. *Journal of Refractive Surgery* is the most prolific journal on myopic corneal refractive surgery research and *Journal of Cataract and Refractive Surgery* is the most influential journal in this field. The priority themes involved wound healing, cytobiology, and biomechanics, which are central to the safety and efficacy of refractive surgery. Taken together, the results of these analyses will help researchers understand the current state of research and provide promising directions for future research. New studies should consider exploring specific aspects of myopic corneal refractive surgery research, such as cytobiology and corneal biomechanics. We aim to study other aspects of myopic corneal refractive surgery using different literature databases and bibliometric methods such as bibliographic coupling analysis. Altmetrics is a new comprehensive bibliometric method used to assess the academic and social impact of research results and can also be applied in conjunction with scientometric analysis to better understand trends and new field research areas.

## Data availability statement

The raw data supporting the conclusions of this article will be made available by the authors, without undue reservation.

## Author contributions

FY and YD performed the bibliometrics analysis and drafted the manuscript. FY and MA organized the manuscript writing. CB oversaw the search strategy. YW reviewed the manuscript. All authors contributed to the article and approved the submitted version.

## Funding

This research was funded by the National Natural Science Foundation of China, grant no: 82271118, and the Young and Middle-Aged Talents Project of Hubei Provincial Department of Education of China, grant no: Q20222112.

## Conflict of interest

The authors declare that the research was conducted in the absence of any commercial or financial relationships that could be construed as a potential conflict of interest.

## Publisher’s note

All claims expressed in this article are solely those of the authors and do not necessarily represent those of their affiliated organizations, or those of the publisher, the editors and the reviewers. Any product that may be evaluated in this article, or claim that may be made by its manufacturer, is not guaranteed or endorsed by the publisher.

## Footnotes

^1^ www.vosviewer.com

^2^ https://www.citnetexplorer.nl/
